# Analysis of Polyphenols During Alcoholic Fermentation of Red Grape Aglianico (*Vitis vinifera* L.): Potential Winemaking Optimization and Pomace Valorization

**DOI:** 10.3390/molecules29245962

**Published:** 2024-12-18

**Authors:** Francesco Errichiello, Martino Forino, Luigi Picariello, Luigi Moio, Angelita Gambuti

**Affiliations:** Department of Agricultural Sciences, Grape and Wine Science Division, University of Naples Federico II, 83100 Avellino, Italy; francesco.errichiello@unina.it (F.E.); luigi.picariello@unina.it (L.P.); moio@unina.it (L.M.); angelita.gambuti@unina.it (A.G.)

**Keywords:** Aglianico wines, waste-to-resource winemaking, wine phenolic recovery, polyphenol release timing

## Abstract

The polyphenol extraction and evolution during a traditional 14-day fermentation of the Aglianico red grape, a variety widely cultivated across Southern Italy, was for the first time investigated, with the purpose of optimizing the phenolic profile in finished wines. Anthocyanins, BSA-reactive tannins, iron-reactive phenols, and vanillin-reactive flavans (VRFs) were analyzed in the free-run must, pressed pomace liquid, and in pomace extracts at different maceration times. Experimental evidence suggested that, instead of the typical 14-day maceration of Aglianico grapes, it is recommendable to choose an 11-day maceration in order to prevent the over-extraction of polyphenols that may detrimentally affect the sensory characteristics of wines. In fact, Aglianico wines, if not properly produced, can be affected by excessive astringency due to its high tannin contents. The findings of the present study can provide insightful knowledge to all the winemakers dealing with grape varieties characterized by high quantities of tannins. Also, an earlier racking would supply grape pomaces extremely rich in valuable phenolic compounds to be extracted and reused in several industrial segments in the frame of circular bioeconomy.

## 1. Introduction

Maceration is a key phase of red winemaking, during which solid components of grapes (skins, seeds, and occasionally stems) are in contact with the must. In this phase, key compounds responsible for some sensory properties of wines, including color, flavor, aroma, and texture, are released into the must [1,2,3]. Among such molecules, polyphenols, extracted from both skins and seeds, determine the wine color, astringency and aging potential. Wines typically show distinct phenolic profiles from both a qualitative and quantitative perspective, as a result of the specific phenolic composition of the grape variety as well as of pedoclimatic conditions. Generally, major classes of polyphenols abundant in red grapes include anthocyanins and proanthocyanidins [4]. Anthocyanins are responsible for the red and purple colors of wines [5,6], and their biosynthesis usually begins in the grape skins at véraison (the onset of ripening) to continue throughout the ripening phase [5,7].

Unlike anthocyanins, which are produced solely in the berry skins, proanthocyanidins, polymers of flavan-3-ol units, constitute another abundant class of polyphenols, and are found in both the vacuoles of skin cells and the inner and outer seed integuments [8]. The structure and size of these compounds, also referred to as tannins, in wine largely vary on the basis of their origin within the grape berry. Skin tannins generally have a higher molecular mass compared to seed tannins [5]. Their molecular sizes deeply impact on the wine astringency on account of their ability to precipitate human salivary proteins [4,9,10]. 

Extraction of polyphenols during fermentation is a complex process influenced by several physical factors, including solvent, temperature, and contact surface area. In general, polyphenol extraction proceeds through the following two major and subsequent mechanisms: an initial rapid release from the edges of broken skin cells, as a consequence of the grape crushing, followed by a slower, concentration-driven diffusion through the solid grape material [11,12]. This process is modeled by Fick’s second law of diffusion, according to which the diffusion rate depends on the solute concentration gradient and the internal diffusion coefficient [13]. 

**Figure 1:**
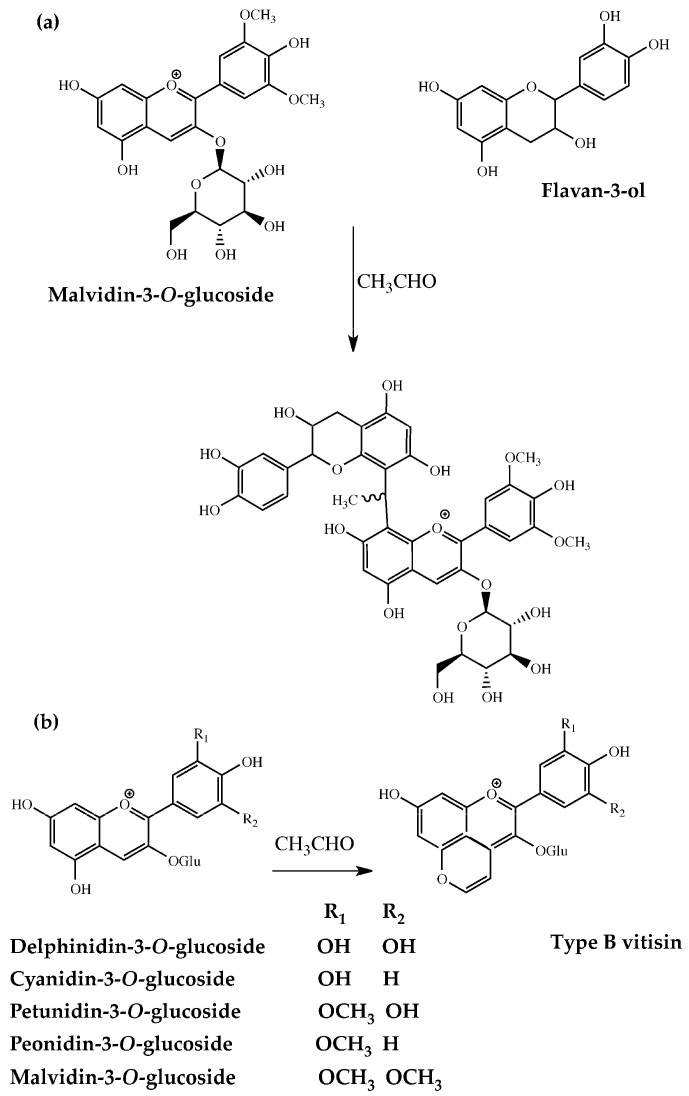
Reaction between catechin and malvidin-3-glucoside in an acid medium, in the presence of acetaldehyde (**a**) and structures of type B vitisins (**b**).

The extraction of polyphenols during maceration is not constant over time. Anthocyanins are mainly extracted during the first few days of maceration and usually peak after three to five days [14,15]. Additionally, during maceration, anthocyanins may undergo partial adsorption, degradation, and primarily polymerization that leads to the formation of pigments [16,17]. These later play a crucial role in the wine color evolution, as they enhance both the product stability and its chromatic complexity over time [18,19]. Acetaldehyde, whose origin can be traced back to either yeast metabolism during early fermentation or to ethanol oxidation, is often involved in the above-reported polymerizations. Notably, acetaldehyde promotes the formation of ethylidene-bridged oligomers along with the so-called pyranoanthocyanins, [9,20] (Figure 1). From a gustative standpoint, ethylidene-bridged compounds exhibit lower perceived astringency than unmodified tannins [4,21].

In regard to proanthocyanidin, their extraction is also a complex process greatly influenced by their chemical nature and molecular size as well as by the composition of the wine matrix [11,22]. In fact, ethanol along with anthocyanins and cell wall material can modulate the extraction and concentration of proanthocyanidins during the maceration phase. 

In addition to anthocyanins and proanthocyanidins, other phenolic compounds, such as flavonols and flavan-3-ols, are extracted during maceration. 

Flavonols, which in grapes exist predominantly in glycosidic forms, are extracted from skins more slowly than anthocyanins, likely on account of their scarce hydrophilicity [23,24]. Since their extraction proceeds at a slower rate, flavonols typically peak after 8–9 days of maceration.

Flavan-3-ols, precursors of proanthocyanidins, are extracted from both skins and seeds, but with different kinetics [25,26,27]. Those present in the skins are rapidly released over the first few days of maceration and reach 80–85% of their maximum concentration usually in five days. Conversely, the extraction of flavan-3-ols from seeds is slower and requires up to ten days or even more.

In this study, we investigated the extraction and evolution of polyphenols during a typical 14-day maceration of Aglianico red grapes. Aglianico is a polyphenol-rich variety used to produce prestigious Italian DOCG (Denominazione di Origine Controllata e Garantita) wines like Taurasi and Aglianico del Taburno. However, this cultivar presents unique challenges on account of its high tannin levels that often make wines excessively astringent. When simulated macerations of Aglianico, Merlot and Cabernet Sauvignon grape skins or seeds were conducted [28], the reactivity of extracts to salivary proteins was highest for the Aglianico seed tannins compared to international varieties. This suggested that the management of solid parts of the berry can strongly affect the sensory quality of the Aglianico wines and, hence, their acceptability by consumers. Nowadays, studies on the impact of maceration conditions on the phenolic composition of the Aglianico wines are quite few. Recently, Giacosa et al. (2023) [29] evaluated the effect of seeds on the anthocyanin extraction from the skins from four Italian red winegrape varieties (Aglianico, Nebbiolo, Primitivo and Sangiovese) and showed interesting differences in terms of anthocyanin extraction rate, content and profile. It follows that, for these grape cultivars, it is necessary to avoid the over extraction of astringent phenolic compounds during maceration. In this context, the Aglianico grape cultivar also emerges as a model for all those red cultivars rich in seed tannins. 

Therefore, a primary goal of our research was to provide, for the first time, a careful understanding of the Aglianico maceration, whose proper management is a prerequisite to obtain high-quality wines with the desired sensory attributes such as color, flavor, and texture. 

A further aim of this study, strictly intertwined with the previous one, was the chemical analysis of the residual phenolic contents of the resulting grape pomace collected from the fermentation tanks at different maceration times. This chemical investigation served the double purpose of evaluating (1) the enological potential of the so-called fermentation cap as a polyphenol reservoir useful to adjust the final phenolic content of the wine; and (2) the potential of grape pomace as a source of valuable compounds as a function of the maceration time. Grape pomace, which alone accounts for about 45% of total winemaking by-products, is in fact a still largely underutilized resource with significant environmental and economic implications for wineries. On account of the paramount quantities of beneficial compounds still present in it, pomace holds great potential to be valorized by recovering valuable molecules. 

## 2. Results and Discussions

### 2.1. Analytical Strategy

Aglianico red grape variety was selected to investigate the extraction of phenolic compounds and their concentration evolution in must throughout a typical 14-day maceration period. To ensure sample homogeneity during maceration, the solid-to-liquid ratio was standardized in each tank for all the biological replicates. We did not use metabisulfite (MBK) in order to prevent any potential interaction with polyphenols [30] as well as with the vacuolar walls of grape skins that would affect the anthocyanin release during maceration [13]. 

With the purpose of providing a comprehensive picture of the phenolic extraction and evolution in must during maceration, we analyzed the following polyphenol classes: iron-reactive phenols, vanillin-reactive flavans (VRFs), BSA-precipitable tannins, polymeric pigments (PPs), and total phenols. To this aim, we resorted to methods commonly used for the determination of the mentioned polyphenol classes, as specified in Section 3. 

The BSA-reactive tannin quantification method suffers from some limitations; for example, monomers and dimers are usually undetected, whereas up to 93% of octamers are indeed revealed [31,32]. Therefore, the actual content of smaller tannins is certainly underestimated. These low-molecular-weight tannins are more reliably quantified by the VRF assay.

Also, we investigated the color characteristics of the must samples, as they are strictly correlated with the polyphenol profile. 

As mentioned in the Introduction, besides studying the extraction and evolution of phenolic compounds during maceration in Aglianico red grape must, we intended to pursue the additional goal of investigating the possibility of recovering valuable compounds from grape pomace. Accordingly, the results and discussion have been divided into the following sections: analysis of polyphenols in the supernatant must (SUR); analysis of polyphenols in liquids obtained (1) by pressing fermentation caps (PREs), the layer of grape skins and seeds that rises to the surface of the fermentation vessel due to the release of carbon dioxide, and (2) by exhaustively extracting residual grape pomace (EXH) with a hydroalcoholic solution (80% ethanol).

### 2.2. Polyphenol Analysis in the Liquid Must (SUR)

The analysis of polyphenolic compounds in SURs during the 14-day maceration period brought to light significant changes in terms of concentrations (Table 1 and Table 2).

Iron-reactive phenols, BSA-reactive tannins and VRFs all increased from d2 to d14 (Table 1). Their concentrations at the end of the maceration had more than doubled. Generally, the greatest increase for iron-reactive phenols (+53.6%) and BSA-reactive tannins (+66.4%) was observed between d2 and d4. From d7 to d9 as well as from d11 to d14, the level of iron-reactive phenols appeared to be approaching a plateau; this observation was likely the consequence of a saturation of the extracting solution and/or of the establishment of an equilibrium between the compounds solubilized into the must and those still contained in the grape solids. Likewise, BSA-reactive tannins stabilized between d11 and d14, thus indicating that their extraction may have peaked around d11. This might be potentially due to either their depletion in grape solids or their interaction with other wine components, such as proteins or polysaccharides, which can limit the tannin release into the must through non-covalent interactions [2]. In fact, high-molecular-weight tannins may form hydrogen bonds and hydrophobic interactions with polysaccharides present in the cell walls of grapes. Additionally, proteins such as expansins, which are rich in hydroxyproline residues, can interact with polymeric tannins, further impacting on their extraction [2]. VRFs reached a plateau between d7 and d9, then significantly increased at d11 (+48%) before stabilizing around d14. This trend can be explained by recalling that flavan-3-ols, primarily located in thin-walled cells situated between the external hydrophobic cuticle and the inner lignified layers, are released from seeds generally more slowly than from berry skins [2].

Iron-reactive phenols, BSA-reactive tannins and VRFs are all responsible for key wine properties. In more detail, iron-reactive phenols are crucial for the oxidative stability of wine on account of their potential to act as pro-oxidants in the presence of metal ions such as iron, which can affect both the color and sensory properties of wines. Thus, by assessing their concentration, winemakers can more effectively manage oxidation processes during vinification, preserving the desired phenolic profile and preventing unwanted oxidative reactions [33]. 

An important sensory attribute whose origin can be traced back already during the maceration is astringency. It plays a fundamental role in the overall taste balance of red wines, contributing to their structure and complexity. Along with astringency, bitterness, although less desired, can influence the perception of the wine balance. In this regard, BSA-reactive tannins are largely responsible for the sensation of astringency [34], while VRFs mainly contribute to bitterness [35]. It follows that reaching an appropriate balance of these classes of compounds during maceration will determine the sensory qualities of the final wine. 

A further class of phenolics analyzed during the fermentation was that constituted by anthocyanins (Table 2). These pigments increased from 269.02 ± 13.13 mg/L (d2) to 452.32 ± 9.72 mg/L (d7) when their concentration reached the maximum level. It remained roughly stable through d11, and then it started to decrease (−14.5%) until d14. The subsequent decline in anthocyanins may be due to different reasons. Among other things, anthocyanins may have been converted into polymeric pigments and/or adsorbed by grape skins, seeds and stalks [2,34,36]. Polymeric pigments, formed by the reaction between anthocyanins and tannins during both alcoholic fermentation and wine aging [37,38], guarantee the desired stabilization of the wine color over time. Under our experimental conditions, polymeric pigments increased from d2 up to d11 to remain constant through the end of the alcoholic fermentation (Table 2). 

As for the chromatic characteristics of must throughout the maceration, the base color parameters CI and hue (Table 2) were measured. CI turned out to be steadily increasing from d2 to d11 when it reached the highest value. No significant differences were observed between d11 and d14. This trend reasonably accounts for the continued extraction of anthocyanins but it is also due to the pigmentation phenomenon that peaks in concomitance with high alcohol contents [39]. 

Conversely, the hue (Table 2) decreased from d2 to d9, thus suggesting a shift towards a more intense red color, commonly defined by CI. After d9, the hue began to increase, consistently with data relative to CI and anthocyanins. Reasonably, the increase in the anthocyanin content initially boosted the color intensity (lower hue values), while subsequent pigment transformations led to a stabler, yet darker and orange appearance in color [40].

**Figure 2:**
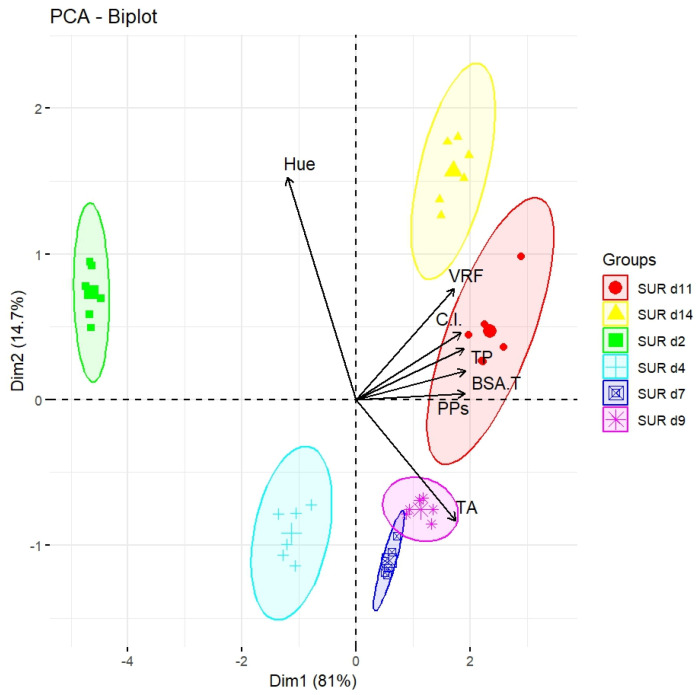
Biplot of six SUR sampling times for the first two principal components (Dim1 and Dim2), extracted by the PCA. Iron-reactive phenols (TP), BSA-reactive tannins (BSA-T), vanillin-reactive flavans (VRFs) and total anthocyanins (TA). The vectors indicate the loadings of the original variables.

Once all of the above-described results were obtained, a principal component analysis (PCA) was applied to identify differences between the various groups (SUR d11, d14, d2, d4, d7, and d9) (Figure 2). The obtained biplot showed how the different groups were distributed along the first two principal components (Dim1 and Dim2), which explain 81% and 14.7% of the total variance in the data, respectively.

Dim1, which explains 81% of the total variance, clearly separated the groups based on the concentration of iron-reactive phenols, vanillin-reactive flavans, BSA-reactive tannins, and polymeric pigments (PPs). In particular, the SUR d11 group (red circles) showed a clear correlation with higher levels of vanillin-reactive flavans, BSA-reactive tannins, polymeric pigments and iron-reactive phenols located towards the upper right quadrant. This group thus appeared to have a specific phenolic composition characterized by high concentrations of the above classes of compounds.

The SUR d14 group (yellow triangles) was also positioned in the upper right quadrant, near the arrow indicating vanillin-reactive flavans, suggesting a similar chemical composition to that of SUR d11. 

On the other hand, the SUR d2 group (green squares) was located in the upper left, associated with negative values on Dim1. This indicated a very different chemical composition from SUR d14 and SUR d11, with a lower presence of reactive phenols and flavanols. 

Dim2, which explains 14.7% of the total variance, further contributed to the separation of the groups, bringing to light differences in terms of anthocyanin concentration. In particular, the SUR d7 and SUR d9 groups (pink stars and purple asterisks, respectively) were associated with high concentrations of anthocyanins, as pointed out by the direction of the anthocyanin arrow, located in the lower part of the biplot.

In conclusion, the PCA biplot provided a clear visualization of how the phenolic- and color-related characteristics evolved throughout the fermentation process. Initially, there was a significant extraction of anthocyanins. Subsequently, a gradual increase in the concentration of ferric-reducing phenolic compounds and tannins was observed through d11. After this time, changes became less pronounced. The inverse relationship between anthocyanins and hue indicated that polymeric pigments played a crucial role in maintaining the overall color stability of the wine during the fermentation process.

### 2.3. Analysis of Polyphenol in the Liquid Obtained by Pressing Wine Fermentation Cap (PRE) 

Starting from the second day (d2) of alcoholic fermentation, we sampled aliquots of PREs. These liquids are known to be rich in phenolic compounds and are commonly reused in winemaking to balance the polyphenolic profile of the wine and, thus, guaranteeing the desired longevity and complexity of long-aging wines [41,42].

Table 3 and Table 4 show the concentrations of phenolic compounds (iron-reactive phenols, BSA-reactive tannins, VRF, total anthocyanins, PPs, CI and hue) in the PREs. The concentration of iron-reactive phenols (Table 3) exhibited a consistent and significant increase from d2 to d14, rising from 1651 ± 149.92 mg/kg (of fresh grape) at d2 to 4086.52 ± 400.96 mg/kg at d14. A substantial increase occurred between d2 and d4, with a 57% rise, indicating an initial rapid release of these compounds from the grape pomace during the early stages of fermentation. This trend mirrors the one observed in SURs, likely due to the initial degradation of the vegetable cell walls and the solvent extraction yield. Between d4 and d7 as well as between d11 and d14, the levels appeared to be stabilized, thus pointing out a plateau in the process. 

BSA-reactive tannins (Table 3) also increased throughout the fermentation, rising from 462.44 ± 10.58 mg/kg at d2 to 1263.55 ± 98.30 mg/kg at d14. The largest increment (42.6%) was observed between d11 and d14. This trend reflected the combined influence exerted by ethanol and by the condition of the vegetable cells, which released high quantities of tannins. 

VRFs (Table 3) showed a general increasing trend during maceration, with concentrations rising from 86.94 ± 3.35 mg/kg at d2 to about 205.35 ± 20.66 mg/kg at d14. A plateau was observed between d4 and d9, followed by a significant increase at d11 (20%) before stabilizing around d14. The initial rapid increase reflected the early release of lower-molecular-weight flavonoids from grape pomace. The plateau between d4 and d9 could be attributed to at least two reasons: the formation of polymeric pigments, which may have reduced the flavonoid concentration [43,44], and the value of the ethanol concentration that was not high enough to allow for the extraction of low-molecular-weight tannins from seeds [45].

In regard to the pigments, the concentration of total anthocyanins in the PREs (Table 4) decreased from d2 to d7 to remain stable from that day onward. No significant differences were detected in the content of polymeric pigments (PPs; Table 4), which remained stable across different time points. Color intensity (Table 4) steadily decreased up to d7 and then increased, while hue increased until d11, indicating a shift towards a more intense yellow color, commonly associated with lower anthocyanin concentrations.

**Figure 3:**
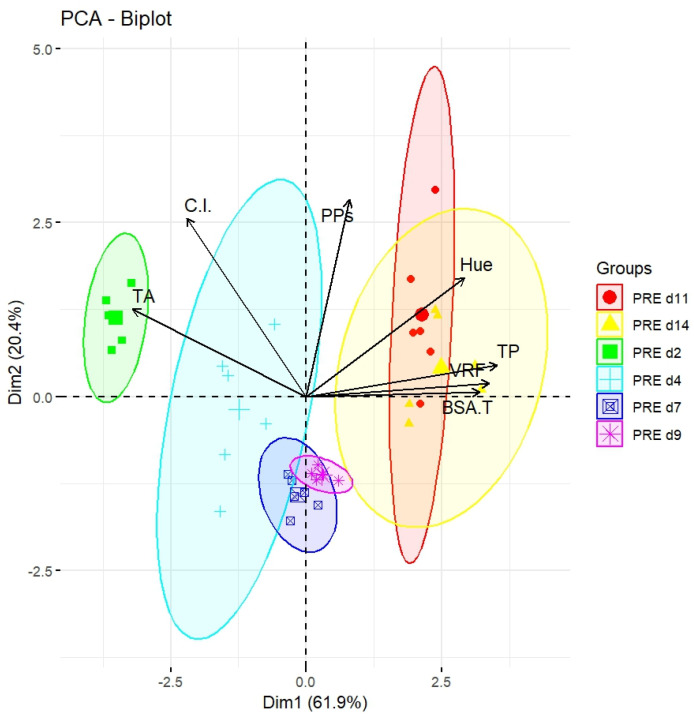
Biplot of six PRE sampling times for the first two principal components (Dim1 and Dim2), extracted by the PCA. Iron-reactive phenols (TP), BSA-reactive tannins (BSA-T), vanillin-reactive flavans (VRFs) and total anthocyanins (TA). The vectors indicate the loadings of the original variables.

To better visualize the above-described data, a PCA analysis was again carried out (Figure 3). Principal Component 1 (Dim1), explaining most of the total variance (61.9%), showed a clear separation of samples based on the concentration of different phenolic compounds. The variables “BSA-reactive tannins,” “Iron-reactive phenols,” “VRF”, and “Hue” were all positively correlated with Dim1, suggesting that these compounds progressively increased during the fermentation process. For instance, samples d11 (red group) and d14 (yellow group) were located at the right, indicating a higher concentration of the above compounds. Conversely, samples d2 (green group) and d4 (light blue group) moved to the left, indicating a lower concentration of the very same compounds at the beginning of the process.

Principal Component 2 (Dim2), explaining 20.4% of the total variance, is mainly influenced by the variables “Anthocyanins,” and “C.I.”. Sample d2 (light green group) was strongly associated with high levels of anthocyanins, while all samples d2, d4, d7, d9, d11 and d14 (light green and blue, blue, pink, red and yellow) showed a greater correlation with PPs. This suggested that there might be no correlation between the quantity of polymeric pigments among the groups.

Samples d2 and d4 appeared to the left of the biplot, reflecting low concentrations of iron-reactive phenols, BSA-reactive tannins, and VRFs at the beginning of fermentation. As time progressed (through d14), samples gradually shifted to the right along Dim1, indicating an increase in the concentration of the above phenolic compounds. This trend reflected the increased phenolic extraction from skins and seeds during maceration.

Samples d11 and d14 were grouped at the right part of the biplot, with an overlap, as a consequence of a similar phenolic and chromatic composition at these two times. This datum confirmed that, after d11, changes in the concentrations of iron-reactive phenols and tannins became less significant, with a resulting stabilization of the phenolic compositions.

PCA highlighted a clear evolution of the phenolic and chromatic composition of the PREs during fermentation. While the variables “C.I.” and “PPs” mainly influenced the separation along Dim2, the anthocyanins, iron-reactive phenols, BSA-reactive tannins, and reactive flavans dominated the variability along Dim1. Samples at later times (d11 and d14) presented a more stable phenolic composition compared to those at earlier times (d2 and d4). 

The highest recoverable quantity of phenols through pressing was detected at around d11, when the levels of iron-reactive phenols and VRFs tended to stabilize. Beyond this time, the concentrations of phenolic compounds did not significantly increase, as confirmed by PCA analysis, which showed a similar phenolic composition and chromatic characteristics of samples at both d11 and d14. 

Therefore, from a technological point of view, we can conclude that it is not advisable to leave the pomace in contact with the must until the end of the alcoholic fermentation. Indeed, should maceration be stopped around d11, the over-extraction of phenolic compounds would be prevented. This is a strategic piece of advice since after d11, in the specific case of Aglianico red grape, there would be an increase in the must of undesirable phenolics that may have a detrimental impact on the astringency of wines and consequently on their quality.

### 2.4. Analysis of Polyphenol Extracted from Residual Grape Pomace (EXH)

Finally, grape pomaces, constituted by the pressed fermentation caps, were extracted with a hydroethanolic mixture, with the purpose of investigating their phenolic contents.

Table 5 and Table 6 show the concentration of phenolic compounds in EXHs at different times during the 14-day maceration. The chemical analysis of the recovered phenols from EXHs clearly demonstrated that an impressive quantity of these compounds was retained by the pomace in comparison with the corresponding levels detected either in the SURs or in the PREs (both expressed per kg of pressed pomace at 1 atm). In more detail, the iron-reactive phenols (Table 5), at d14, were 12,149.15 ± 832.69 mg/kg in EXHs, a concentration nearly three times higher than that detected in the PREs at the same day. Likewise, BSA-reactive tannins and VRFs were also detected at quite high levels in the EXHs.

The content of total anthocyanins (Table 6) was higher in the EXHs at d2 and decreased until d14; no differences emerged between d11 and d14. On the other hand, PPs (Table 6) decreased at d7 and d11 and slightly increased afterward.

Given the remarkable amount of phenolic compounds retained by pomace, even during the late stages of maceration, potential recovery and reutilization of such valuable molecules is certainly an issue worth investigating. This goal could be achieved by resorting to green methodologies such as the employment of green solvents, dimethyl carbonate and 2-methyl tetrahydrofuran [46], or by Natural Deep Eutectic Solvents (NADESs) that have been proven to efficiently extract bioactive compounds from several organic matrices, with low environmental impact.

### 2.5. Evolution of Phenol Extraction During Maceration

**Figure 4:**
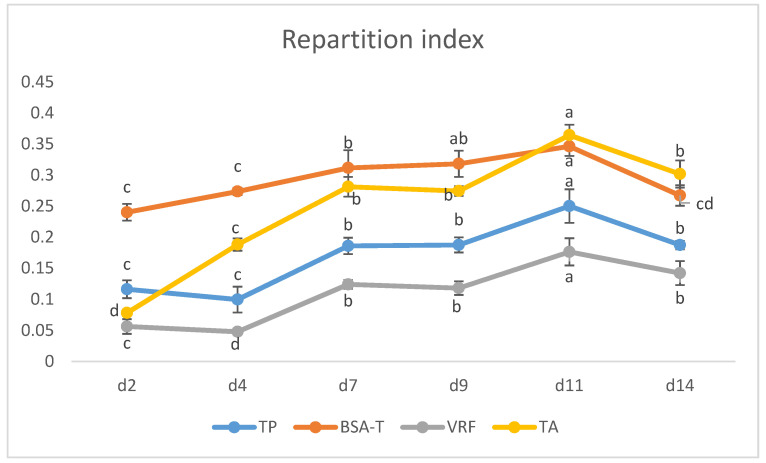
Repartition index of the iron-reactive phenols (TP), BSA-reactive tannins (BSA-T), vanillin-reactive flavans (VRFs) and total anthocyanins (TA) during the 14-day maceration period of the alcoholic fermentation. The repartition index indicates the distribution of these compounds in the must over time, with higher values reflecting greater concentrations of the compounds in the must. Mean values (n = 6) for each phenolic class with different letters are statistically different at (*p* < 0.05).

In order to obtain a more insightful picture of the extraction of polyphenols during the alcoholic fermentation, we rearranged all of the obtained data by creating a phenol repartition index (Figure 4). This index was elaborated by taking into account all the analyzed phenolic classes. Higher values of the index were associated with higher concentrations of phenols in the analyzed must. The ultimate aim of the index was to suggest the most appropriate time to stop the maceration phase.

Figure 4 shows the repartition index of iron-reactive phenols (TP), BSA-reactive tannins (BSA-T), VRF, and total anthocyanins (TA). During maceration, all phenolic classes were progressively extracted up to d11; after that time, a decline was observed. Notably, compared to the other phenolics, anthocyanins (Figure 4) exhibited the fastest rate of extraction. In particular, from d2 to d7, anthocyanins increased more sharply than the other phenolics. This rapid extraction reached its peak at d11 and decreased from that day onwards. The greater speed of anthocyanin extraction confirmed that their solubilization occurs earlier and faster than that of other phenolic compounds. It is also worth noting that in the early stages (d2 and d4), anthocyanins are extracted less efficiently than tannins. This has significant enological implications, particularly if techniques for managing the cap are employed to enhance the transfer from pomace to must with the purpose of appropriately tailoring the desired phenolic profile of the finished wine.

**Figure 5:**
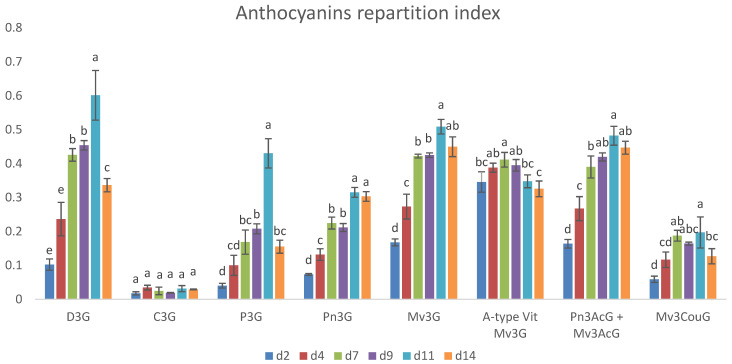
Repartition index of the native anthocyanins during the 14-day maceration period of alcoholic fermentation. D3G: delphinidin-3-*O*-glucoside; C3G: cyanidin-3-*O*-glucoside; P3G: petunidin-3-*O*-glucoside; Pn3G: peonidin-3-*O*-glucoside; Mv3G; malvidin-3-*O*-glucoside; A-type Vit Mv3G: A-type vitisin of malvidin-3-*O*-glucoside; Pn3AcG + Mv3AcG: sum of peonidin-3-*O*-acetylglucoside and malvidin-3-*O*-acetylglucoside; Mv3CouG: *cis* and *trans* malvidin-3-*O*-coumaroylglucoside. Mean values (n = 6) for each phenolic class with different letters are statistically different at (*p* < 0.05).

As described above, monomeric anthocyanins started to decrease after d11 likely due to a resorption by grape pomace and/or to their transformation into polymeric pigments. To obtain a more detailed understanding of the anthocyanin decrease, we calculated the partition indexes for the native anthocyanins (delphinidin-3-*O*-glucoside, cyanidin-3-*O*-glucoside, petunidin-3-*O*-glucoside, peonidin-3-*O*-glucoside, and malvidin-3-*O*-glucoside) along with those relative to common anthocyanin derivatives, including vitisin A, malvidin-3-*O*-coumaroylglucoside, and both malvidin-3-*O*-acetylglucoside and peonidin-3-*O*-acetylglucoside (Figure 5). These latter two compounds were evaluated as a sum, since their chromatographic peaks were partially overlapping and prevented a reliable quantitation of the single compounds.

Our results indicated that delphinidin-3-*O*-glucoside, petunidin-3-*O*-glucoside, and malvidin-3-*O*-coumaroylglucoside exhibited a pronounced decrease following their maximum extraction. Similarly, peonidin-3-*O*-glucoside, malvidin-3-*O*-glucoside, and the combined acetylated anthocyanins also reached their highest concentrations at d11 but did not significantly decrease at d14. Cyanidin-3-*O*-glucoside, in contrast, remained roughly stable over time, which is notably different from the behavior of other anthocyanins. Conducting a study on this molecule in a model solution during the extraction phase would be particularly interesting to understand the mechanisms behind its stability and to determine if specific interactions or environmental factors can somehow contribute to its unique behavior. Vitisin A, although initially present in substantial quantities, showed a marked decline after d9.

In the literature, it is reported that during the late stages of vinification, anthocyanin concentrations tend to decrease after reaching a maximum level. There could be several causes of such a decrease, including interactions with yeast cell walls and precipitation with tartaric salts [47,48]. In particular, it is well known that yeast cell walls, composed of mannoproteins, polysaccharides, glucans, and chitins, are able to adsorb anthocyanins based on their polarity and hydrophobic or hydrophilic nature [49]. Different yeast strains have been shown to impact anthocyanin profiles during fermentation [50,51]. In particular, acylated anthocyanins are more strongly adsorbed than non-acylated forms, while pyranoanthocyanins like vitisins are adsorbed to a lesser extent [52,53]. Also, the grape pomace, rich in fibrous material, can likely interact with anthocyanins, further intensifying their decline in musts [54].

Identifying the optimal extraction time for specific phenolic classes can be crucial when designing wines with different aging potentials. In the case of red wine intended for long aging, winemakers should aim at maximizing the extraction of tannins and anthocyanins, specifically BSA-reactive tannins and anthocyanins like malvidin-3-*O*-glucoside. Our data suggested that extending the contact time between the must and the grape pomace up to d11 would allow for the enrichment of the must of necessary phenolic compounds such as proanthocyanidins and total anthocyanins. Even if anthocyanins begin to decline after d11, a final desired concentration can be obtained by appropriately managing the fermentation cap especially over the initial days of maceration. Conversely, a wine intended to be consumed soon is expected to feature freshness and an easy-drinking nature. In this case, a softer tannic structure and a more approachable profile are desirable; hence, an early separation of the solids during maceration might be a preferable strategy. In regard to the Aglianico wines, winemakers might choose to separate the must from the pomace before the phenolic extraction reaches its peak at d11. This would result in a wine with a lower tannin content, yielding a smoother, fruit-forward wine suitable for early consumption. 

Finally, our findings have an important impact on Aglianico winemaking as they provide enologists with strategic knowledge indispensable for the proper management of this challenging grape variety, which is characterized by elevated tannin contents. This study has proven that the traditional 14-day maceration period is somehow too long, as it would cause the over-extraction of unnecessary and even detrimental polyphenols. Besides Aglianico, other grape varieties, including Nebbiolo, Sagrantino and Tannat [55,56] among others, are characterized by high tannin levels. Therefore, results from this study could benefit the winemaking practices relative to the mentioned grapes as well. 

Another relevant aspect to emphasize is that an earlier end of the Aglianico fermentation would supply pomaces richer in polyphenols. Therefore, by means of environmentally friendly technology, polyphenols could be extracted with the purpose of reusing them in a broad range of industries. As a way of example, in food industries, the growth of harmful bacteria could be prevented thanks to the antimicrobial properties of phenols. The possibility to develop the formulation of functional foods enriched with polyphenols extracted from organic matrices has been also explored. Anthocyanins and tannins have been successfully employed to produce biodegradable films for food packaging or as natural dyes in the textile sector, without mentioning the well-known use of polyphenols in the pharmaceutical segment on account of their many health-related properties [57]. Recently, the cosmetic potential of traditional and even fungus-resistant grape varieties has also been interestingly assessed [58].

Definitively, further scientific research and technological advancements are necessary to address the need for optimized formulations (i.e., powders, capsules, beverages) that are somehow stable and marketable. In this framework, even legal issues are to be considered in terms of compliance with safety, labelling, and quality control standards. It follows that many multidisciplinary aspects remain to be accurately studied and investigated. 

## 3. Materials and Methods

### 3.1. Chemicals

All solvents were of HPLC grade or higher. The following chemicals were purchased from J.T. Baker (Levanchimica, Bari, Italy): glacial acetic acid, hydrochloric acid, methanol, acetonitrile, ethanol, sodium dodecyl sulfate (SDS), triethanolamine, iron chloride, vanillin, tartaric acid, formic acid, sulfuric acid, 2,4-dinitrophenylhydrazine, sodium hydroxide, bovine serum albumin (BSA), and malvidin-3-*O*-glucoside. Water was purified using a Milli-Q system from MilliporeSigma (Burlington, MA, USA). The yeast strain used in this study was Zymaflore FX10, supplied by Laffort Oenologie (Bordeaux, France) along with Thiazote^®^PH obtained from the same supplier.

### 3.2. Red Wine Fermentation Trial

Aglianico red grapes were obtained from the Taurasi DOCG region in Campania, Italy. These grapes, supplied by the Quintodecimo winery in Mirabella Eclano, were harvested in 2022. The timing of the harvest was determined by monitoring the sugar levels during grape ripening. Harvest occurred at the maturity stage when the sugar concentration in the berries turned out to be constant over a period of one week (23 ± 0.3 °Brix). A total of 50.0 kg of healthy grapes was manually picked to ensure a representative sample. The grapes were then mechanically crushed and destemmed. To standardize the solid-to-liquid ratio, the must was separated from the skins using a steel sieve. Three fermentation tanks were separately filled with 8.6 L of must and 6.5 kg of skins and seeds, as to ensure the same ratio in each tank. To avoid any potential interaction with polyphenols, metabisulfite was not used in this study.

Fermentation occurred at a controlled temperature of 21 ± 1 °C following the inoculation with 20 g/hL of FX10 Zymaflore yeast. The fermentation cap was immersed twice daily, and the fermentation progress was tracked by monitoring soluble solid contents. In more detail, the must was subjected to daily pump-overs, during which the containers were manually agitated three times. Midway through fermentation, a nitrogen supplement was added to all samples: 30 g/hL of Thiazote^®^ (containing ammonium phosphate and thiamine) was used to support yeast growth. Upon completion of fermentation, the wines were decanted, cold-stabilized at −6 °C, and then stored.

### 3.3. Sampling Procedures and Experimental Plan 

Sampling was carried out over a 14-day maceration period, during which 6 samples (6 SURs, 6 PREs, and 6 EXHs, respectively) were collected for each time point. Samples of the free must (SUR) were collected on the following days: day 2 (d2), day 4 (d4), day 7 (d7), day 9 (d9), day 11 (d11), and day 14 (d14). These time intervals were chosen on the basis of our knowledge of the Aglianico maceration, during which alcoholic fermentation does not undergo significant changes throughout the middle phase. Following each sampling of SUR, an aliquot of the collected pomace was pressed at 1.0 bar to obtain the resulting liquid (PRE samples). Once pressed, the pomace was subjected to an exhaustive extraction by a hydroethanolic mixture (80% EtOH) to produce the EXH samples. 

### 3.4. Wine Color Evaluation and Spectrophotometry-Based Analyses of Phenols

Wine color intensity (CI) of the SUR and PRE samples was assessed by measuring the cumulative absorbance at 420 nm (yellow), 520 nm (red), and 620 nm (blue), while hue was determined as the ratio of absorbance at 420 nm to 520 nm, following the Glories method [59].

In all samples, vanillin-reactive flavanols (VRFs) were quantified according to the procedure described by Di Stefano and Guidoni [60]. A test tube was prepared by diluting (1:10) the wine with pure methanol. In a subsequent step, 125 μL of the diluted wine was mixed with 750 μL of a 4% vanillin solution in methanol and 375 μL of concentrated hydrochloric acid (12.01 M). After a 15 min incubation in cold water and a further 15 min incubation at room temperature, absorbance was recorded at 500 nm. Likewise, a blank was prepared replacing the vanillin solution with pure methanol. The concentration of VRFs was calculated as (+)-catechin equivalents (mg/L) using a calibration curve with a linear range of 2.0–250.0 mg/L. The calibration parameters were as follows: 0.02 < ΔE < 0.05 (slope 277.26, intercept −3.58); 0.05 < Δ < 0.18 (slope 250, intercept −3.25); and 0.18 < Δ < 0.83 (slope 314.23, intercept −12.91).

The total anthocyanins, BSA tannins, total phenolics, small polymeric pigments (SPPs), and large polymeric pigments (LPPs) were quantified by the assay developed by Harbertson [35]. This method involves protein precipitation with bovine serum albumin (BSA) and bisulfite bleaching to differentiate between two categories of polymeric colors in wine (PPs): LPPs, which precipitate with proteins, and SPPs, which remain in solution. Chromatic measurements were performed using a 7305 spectrophotometer (Jenway Bibby Scientific Limited, Stone, UK) with 10 mm plastic cuvettes. Each analysis was performed with three experimental and two analytical replicates.

### 3.5. High-Performance Liquid Chromatography (HPLC) Analyses of Anthocyanins

In all samples, monomeric anthocyanins were chromatographically identified by using the OIV method of analysis [61] partly modified as reported by Errichiello [62]. The HPLC system (Agilent 1260 Infinity II LC system—Santa Clara, CA, USA) included a binary pump, a two-channel degasser, a diode array detector (DAD, G7114A), and a manual injector with a 20 μL loop. Chromatographic separation was achieved with a C18 column, Synergi 4 μm Fusion—RP 80 (250 × 3.0 mm, 4 μm particle size) and a pre-column, operating at 40 °C.

Samples were filtered through 0.45 μm Durapore membrane filters (Millipore, Tullagreen. Carrigtwohill, County Cork, Ireland) into glass vials before injection into the HPLC system. Detection was performed by monitoring absorbance at 518 nm [61], and data were processed using OpenLAB CDS ChemStation Edition software C.01.01 (Agilent Technologies, Santa Clara, CA, USA). Elution was conducted at a flow rate of 0.8 mL/min with the following eluents: Solution A, a mixture of milli-Q water (Sigma Aldrich, Milan, Italy), formic acid (Sigma Aldrich ≥ 95%), and acetonitrile (Sigma Aldrich ≥ 99.9%) in a 96:1:3 (*v*/*v*/*v*) ratio; and Solution B, a mixture of water, formic acid, and acetonitrile in a 49:1:50 (*v*/*v*/*v*) ratio.

The gradient program was as follows: initially 94% A and 6% B, transitioning to 70% A and 30% B after 15 min, then to 50% A and 50% B at 30 min, followed by 40% A and 60% B at 35 min, and finally returning to 94% A and 6% B at 41 min until the end of the analysis. Calibration was performed using an external standard method with a calibration curve based on the peak area of malvidin-3-*O*-glucoside. Concentrations were reported as equivalents of malvidin-3-*O*-glucoside (mg/L). All experiments were conducted in duplicate with two analytical replicates. The calibration curve was expressed by the equation y = 5 × 10^−6^x, with an R^2^ value of 0.999, a limit of quantitation (LOQ) of 2 mg/L, and a limit of detection (LOD) of 0.6 mg/L.

### 3.6. Phenolic Evolution Index 

To assess the evolution of phenolic compounds during the 14-day maceration period, a specific index was calculated. This index relates the quantity of phenols present in the SURs to the total quantity of phenols extracted from the pomace, both through pressing (PRE) and through extraction with the hydroalcoholic mixture (EXH). The index is thus expressed as the ratio between SUR phenols and the sum of SUR, EXH, and PRE phenols:

Index = Phenols SUR/(Phenols SUR + Phenols EXH + Phenols PRE)(1)

A high index value is indicative of a greater increase in phenols into the must during fermentation, while a low value indicates a greater decrease in these compounds in the pomace.

### 3.7. Statistical Analysis

Statistical analyses were conducted using IBM SPSS Statistics (version 29.0.1.0). Differences between groups were evaluated using one-way ANOVA, provided that the data met the assumptions of normality and homogeneity of variance. If these assumptions were not met, the non-parametric Kruskal–Wallis test was applied. Statistical significance was set at *p* < 0.05. To study the differences in the measured spectrophotometric parameters, a PCA was performed using R running under Rstudio (Version R-4.3.3, R Foundation for Statistical Computing, Vienna, Austria) (R Core Team 2019). As inputs, seven variables were used: PPs (polymeric pigments), total anthocyanins, VRFs (vanillin-reactive flavans), BSA-reactive tannins, iron-reactive phenols, CI (color intensity) and hue (tonality). The PCA and the biplot of the main results were performed using the R package “factoextra” (version 1.0.7). All analyses were performed in six replicates, which consisted of three different experimental samples (each corresponding to a separate fermentation tank) × two analytical replicates per sample. Mean values along with standard deviations were calculated.

## 4. Conclusions

For the first time, an in-depth analysis of the polyphenol extraction from Aglianico red grapes during a typical 14-day maceration was conducted. Our data suggested that the extraction of iron-reactive phenols, BSA-reactive tannins and vanillin-reactive flavonoids followed an initial rapid phase and reached a plateau after eleven days. Regarding anthocyanins, their concentration peaked after seven days, followed by a sharp decline. 

Hence, with the purpose of producing a wine to be shortly consumed, maceration should be stopped before day eleven (preferentially between day seven and nine) to guarantee freshness and an easy-drinking nature. Conversely, it is advisable to extend maceration up to day eleven for a long-aging wine. This will allow for the extraction of the appropriate amount of phenols involved in the formation of key polymeric pigments that would endow the wine with long-term color stability, flavor and texture. At the same time, an eleven-day maceration would prevent the over-extraction of unnecessary and detrimental phenols, while supplying winemakers with residual grape pomaces extremely rich in valuable compounds. An appropriate recovery of these molecules will ultimately favor the sustainability of wineries.

## Figures and Tables

**Table 1:** Iron-reactive phenols, BSA-reactive tannins and vanillin-reactive flavans released in the liquid must during the 14-day maceration period of the alcoholic fermentation.

	Iron-Reactive Phenols				BSA-Reactive Tannins				Vanillin-Reactive Flavans			
SUR d2	707.13	±	60.49	d	173.62	±	7.37	e	23.15	±	3.96	d
SUR d4	1086.70	±	78.70	c	288.93	±	9.54	d	33.19	±	1.60	c
SUR d7	1350.81	±	60.43	b	333.88	±	12.10	c	46.20	±	4.61	b
SUR d9	1419.86	±	52.12	b	368.72	±	11.51	b	45.96	±	2.23	b
SUR d11	1603.61	±	51.24	a	392.07	±	15.71	a	68.04	±	10.61	a
SUR d14	1673.77	±	92.30	a	412.47	±	11.84	a	65.77	±	9.12	a

Values are expressed as the means ± SD over six replicates (mg/L). Values with different letters along a column, within each sampling time, are statistically different at (*p* < 0.05).

**Table 2:** Total anthocyanins, sum of polymeric pigments (PPs), color intensity and hue released and detected in the liquid must during the 14-day maceration period of the alcoholic fermentation.

	Total Anthocyanins				Sum of PPs				Color Intensity				Hue			
SUR d2	269.02	±	13.13	c	0.40	±	0.04	d	4.37	±	0.03	d	0.78	±	0.01	a
SUR d4	389.57	±	20.47	b	0.81	±	0.07	c	5.37	±	0.17	c	0.66	±	0.01	d
SUR d7	452.32	±	9.72	a	1.13	±	0.04	b	5.48	±	0.10	c	0.64	±	0.01	e
SUR d9	451.70	±	12.00	a	1.10	±	0.06	b	6.20	±	0.14	b	0.64	±	0.00	e
SUR d11	458.68	±	8.39	a	1.39	±	0.14	a	6.58	±	0.31	ab	0.68	±	0.01	c
SUR d14	391.94	±	3.51	b	1.22	±	0.10	a	6.61	±	0.12	a	0.72	±	0.01	b

Values are expressed as the means ± SD over six replicates. Total anthocyanins are expressed as mg/L, whereas color intensity, sum of PPs and hue are in absorbance units (Abs). Values with different letters along a column, within each sampling time, are statistically different at (*p* < 0.05).

**Table 3:** Iron-reactive phenols, BSA-reactive tannins and vanillin-reactive flavans present in the PREs during the 14-day maceration period.

	Iron-Reactive Phenols				BSA-Reactive Tannins				Vanillin-Reactive Flavans			
PRE d2	1651.23	±	149.92	d	462.44	±	10.58	c	86.94	±	3.35	c
PRE d4	2600.40	±	138.28	c	627.31	±	164.78	b	151.87	±	22.84	b
PRE d7	2441.73	±	83.39	c	799.46	±	97.65	b	130.61	±	11.11	b
PRE d9	3140.18	±	80.91	b	776.58	±	123.72	b	153.68	±	2.56	b
PRE d11	3910.89	±	39.00	a	886.20	±	82.47	b	184.50	±	17.95	a
PRE d14	4086.52	±	400.96	a	1263.55	±	98.30	a	205.35	±	20.66	a

Values are expressed as the means ± SD over six replicates (mg/Kg of pomace). Values with different letters along a column, within each sampling time, are statistically different at (*p* < 0.05).

**Table 4:** Total anthocyanins, sum of polymeric pigments (PPs), color intensity and hue present in the PREs during the 14-day maceration period.

	Total Anthocyanins				Sum of PPs				Color Intensity				Hue			
PRE d2	288.00	±	26.67	a	0.73	±	0.14	a	0.43	±	0.01	a	0.31	±	0.00	d
PRE d4	225.53	±	13.64	b	0.74	±	0.20	a	0.34	±	0.03	b	0.28	±	0.03	e
PRE d7	153.84	±	8.47	cd	0.64	±	0.08	a	0.27	±	0.01	d	0.32	±	0.01	c
PRE d9	162.58	±	2.59	c	0.58	±	0.02	a	0.29	±	0.00	c	0.33	±	0.01	b
PRE d11	142.88	±	6.78	d	0.97	±	0.27	a	0.31	±	0.02	b	0.43	±	0.02	a
PRE d14	162.98	±	20.03	cd	0.71	±	0.12	a	0.33	±	0.03	b	0.41	±	0.03	a

Values are expressed as the means ± SD over six replicates. Total anthocyanins are expressed in mg/Kg of pomace, whereas color intensity, sum of PPs and hue are expressed in absorbance units (Abs). Values with different letters along a column, within each sampling time, are statistically different at (*p* < 0.05).

**Table 5:** Iron-reactive phenols, BSA-reactive tannins and vanillin-reactive flavans present in the EXHs during the 14-day maceration period.

	Iron-Reactive Phenols				BSA-Reactive Tannins				Vanillin-Reactive Flavans			
EXH d2	1158.83	±	685.40	c	6063.56	±	476.97	b	837.73	±	38.99	b
EXH d4	27,513.37	±	3126.95	a	8772.25	±	268.69	a	1538.69	±	79.35	a
EXH d7	11,463.78	±	1394.68	c	3770.57	±	153.05	d	642.31	±	75.28	c
EXH d9	17,713.85	±	918.96	b	6067.92	±	619.10	b	832.49	±	118.79	b
EXH d11	8899.53	±	2649.36	c	4010.90	±	298.61	d	720.15	±	45.86	bc
EXH d14	12,149.15	±	832.69	c	4679.75	±	310.43	c	712.74	±	80.40	bc

Values are expressed as the means ± SD over six replicates (mg/kg of EXHs). Values with different letters along a column, within each sampling time, are statistically different at (*p* < 0.05).

**Table 6:** Total anthocyanins, sum of polymeric pigments (PPs), color intensity and hue present in the EXHs during the 14-day maceration period.

	Total Anthocyanins				Sum of PPs				Color Intensity				Hue			
EXH d2	8237.62	±	323.76	a	19.73	±	0.63	a	54.50	±	2.70	a	0.96	±	0.04	b
EXH d4	4215.16	±	313.90	b	18.81	±	1.60	a	49.14	±	2.76	b	1.14	±	0.11	ab
EXH d7	2940.15	±	244.94	cd	15.01	±	0.69	d	32.29	±	0.11	e	1.28	±	0.05	ab
EXH d9	3009.97	±	187.81	c	16.57	±	0.34	b	36.31	±	0.41	cd	1.30	±	0.03	ab
EXH d11	2490.25	±	139.19	d	15.90	±	0.21	c	35.49	±	0.80	d	1.33	±	0.04	a
EXH d14	2442.29	±	321.90	d	17.69	±	0.62	a	37.57	±	0.84	c	1.28	±	0.09	ab

Values are expressed as the means ± SD over six replicates. Total anthocyanins are expressed in mg/kg of EXHs, whereas color intensity, sum of PPs and hue are expressed in absorbance units (Abs). Values with different letters along a column, within each sampling time, are statistically different at (*p* < 0.05).

## Data Availability

The raw data supporting the conclusions of this article will be made available by the authors on request.
